# Quantitative Proteomic Analysis of Primitive Neural Stem Cells from LRRK2 G2019S-Associated Parkinson’s Disease Patient-Derived iPSCs

**DOI:** 10.3390/life10120331

**Published:** 2020-12-07

**Authors:** Hyuna Sim, Ji-Hye Seo, Jumi Kim, Minyoung Oh, Joo-Eun Lee, Areum Baek, Seo-Young Lee, Sun-Ku Chung, Mi-Young Son, Jung-Il Chae, Young-Joo Jeon, Janghwan Kim

**Affiliations:** 1 Stem Cell Convergence Research Center, Korea Research Institute of Bioscience and Biotechnology (KRIBB), Daejeon 34141, Korea; hyunasim@kribb.re.kr (H.S.); omy94107@kribb.re.kr (M.O.); jooeunlee@kribb.re.kr (J.-E.L.); areumbaek@kribb.re.kr (A.B.); myson@kribb.re.kr (M.-Y.S.); 2 Department of Functional Genomics, KRIBB School of Bioscience, University of Science and Technology, Daejeon 34113, Korea; 3 Department of Dental Pharmacology, School of Dentistry, BK21 Plus, Jeonbuk National University, Jeonju 54896, Korea; wlgpsid7156@naver.com (J.-H.S.); returnjumi@gmail.com (J.K.); jichae@jbnu.ac.kr (J.-I.C.); 4 Division of Herbal Medicine Research, Korea Institute of Oriental Medicine (KIOM), 1672 Yuseong-daero, Yuseong-gu, Daejeon 34054, Korea; 09seoyoung03@kiom.re.kr; 5 Division of Clinical Medicine, Korea Institute of Oriental Medicine (KIOM), 1672 Yuseong-daero, Yuseong-gu, Daejeon 34054, Korea; skchung@kiom.re.kr

**Keywords:** Parkinson’s disease, pNSCs, LRRK2, proteomic analysis, Ku80, DNA damage response

## Abstract

Parkinson’s disease (PD) is a common neurodegenerative disease, causing movement defects. The incidence of PD is constantly increasing and this disease is still incurable. Thus, understanding PD pathophysiology would be pivotal for the development of PD therapy, and various PD models have thus been already developed. Through recent advances in reprogramming techniques, a primitive neural stem cell (pNSC) derived from PD patient induced pluripotent stem cells (iPSCs) could be potentially used as a reproducible and reliable experimental system to analyze the effect of the leucine-rich repeat kinase 2 *G2019S* mutation (LK2GS) in neural cells. Here, we investigated the advantages of such a model system through quantitative proteomic analysis of pNSCs from normal control iPSCs and familial PD patient iPSCs harboring LK2GS. We confirmed that the expression of molecules known to be involved in PD pathogenesis, such as oxidative stress-, cell adhesion-, and cytoskeleton-related proteins, were altered in the LK2GS pNSC. In addition, we showed that down-regulation of Ku80, which was found in the proteomic analysis with LK2GS pNSCs, resulted in apoptosis induced by DNA damage response. Taken together, we suggest that pNSCs from PD iPSCs could provide a reliable and useful model system to study PD. Moreover, the highly expandable pNSC is suitable for multi-omics approaches to understand PD pathologies and discover therapeutic targets for PD.

## 1. Introduction

Parkinson’s disease (PD) is a common progressive and neurodegenerative movement disorder, with a prevalence of more than 1% beyond the age of 65 years [1]. The PD pathological hallmarks include the age-dependent loss of dopaminergic neurons in the substantia nigra pars compacta region in the brain and the progressive spatiotemporal distribution of Lewy bodies and Lewy neurites [2]. Typical PD symptoms include motor features (postural disturbances, bradykinesia, rigidity, or tremor) and non-motor features (hyposmia, sleep disorders, or autonomic-, neuropsychiatric-, and sensory symptoms) [3]. Most PD cases occur sporadically, whereas 5–10% of the cases are inherited with mutations identified in several genes [4]. The age at onset, progression rate, and PD severity vary, which might result from the interaction between environmental factors and genetic mutations [5]. However, the real PD etiology and pathogenesis are still unclear. Until now, PD incidence increases with the aging of the population, just as well as global PD prevalence. Although several studies have been already reported that focus on PD diagnosis and treatment, useful PD model systems are still in demand.

Missense mutations in the leucine-rich repeat kinase 2 gene (LRRK2) locus are the most common known causes of the late-onset familial and sporadic PD forms [6]. LRRK2 protein structure contains a combination of guanosine triphosphatase (GTPase), kinase, and scaffolding domains. The pathological functions of LRRK2 are mainly associated with its aberrant kinase activity [7]. A common LRRK2 genetic mutation is associated with PD pathological features, including dopaminergic neuronal cell death, impaired dopamine neurotransmission, protein synthesis/degradation defects, and oxidative stress [6,7]. The most frequent LRRK2 mutation is c.6055G>A, which results in the substitution of a glycine with a serine in the position 2019 (p.G2019S), situated in the kinase domain. This mutation has been associated with α-synuclein accumulation, mitochondrial dysfunction, and impaired dopamine signaling in the human brain, eventually resulting in the progressive loss of dopaminergic neurons [8,9,10]. Therefore, the LRRK2 G2019S (LK2GS) mutation might be an appropriate genetic mutation to introduce into a novel PD disease model system.

The generation of model systems that accurately recapitulate the LK2GS-associated disease state for PD studies is particularly challenging. For example, animals with genetic mutations that mimic the familial form of parkinsonism, including the LK2GS, fail to show clear evidence of progressive dopaminergic neuron loss or Lewy body formation [11,12,13,14]. Differentiating induced pluripotent stem cells (iPSCs) into dopaminergic neurons was another research approach to introduce novel PD model systems. However, these cells also exhibit variable levels of dopaminergic neuronal toxicity, although other PD pathology features, including Lewy body aggregates, are not as prominent in this system as in the human brain, which could be due to the fact that these systems are generally immature [15,16]. Moreover, such differences might be due to differences between the species and model systems that do not reflect PD characteristics. To better understand PD pathogenesis, we opted to use a human-derived primitive neural stem cell (pNSC). It can be cultured relatively constant and reproducible compared to conventional NSCs, which are changed from neurogenic to gliogenic within several passages [17]. Of note, pNSCs can be generated from iPSCs through a well-established differentiation protocol, and show excellent disease phenotype reproducibility in vitro [18]. Therefore, the LK2GS-pNSCs could potentially provide an unlimited supply of cells for biochemical studies and drug screening.

In this study, we applied a proteomic approach using PD patient-derived pNSCs, harboring the LK2GS mutation, to identify and address the disease-relevant differences between their protein expression profiles and those of healthy controls (WT). Although proteomic analysis has been used as a powerful tool to understand the effector mechanisms of diverse biological processes, few studies have been published that describe PD-related proteomic analyses, and none of those has focused on pNSCs differentiated from the iPSCs derived from PD patients harboring *LRRK2 G2019S* mutation. To identify and characterize the changes of proteome profiles in LK2GS-pNSC compared with WT-pNSC, we carried out comparative proteome analyses using liquid chromatography with tandem mass spectrometry (LC-MS/MS) on differentially expressed proteins (DEPs) in each sample. The DEPs identified in our study act as crucial regulators in oxidative stress-, cell adhesion-, cytoskeleton-, and double-strand break (DSB)-associated proteins, which are known to be related to PD pathologies. We demonstrated that the LK2GS mutation induced DNA damage, increased oxidative stress, and resulted in apoptotic cell death in pNSCs. Therefore, we propose that LK2GS-pNSCs could serve as a unique in vitro cellular disease model to better understand the effect of LK2GS mutation which found frequently in PD patients.

## 2. Materials and Methods

### 2.1. Human-Induced Pluripotent Stem Cell (iPSC) Culture

Human wild type (WT) iPSCs (HPS0076) were purchased from the Riken Cell Bank (Tsukuba, Ibaraki, Japan). Somatic cells from patients with PD (ND14317, ND38262) carrying the LRRK2 G2019S mutation (LK2GS) were purchased from the Coriell Institute for Medical Research (Supplementary Table S1). Somatic cells were reprogrammed by electroporation with episomal iPSC reprogramming vectors as described previously [19,20]. The 3.14 iPSC colonies per 100,000 cells (efficiency 0.003%) were generated. Established iPSCs were cultured on Geltrex-coated culture dishes and fed with TeSR^TM^-E8^TM^ (STEMCELL Technologies, Vancouver, BC, Canada).

### 2.2. Differentiation of iPSCs into pNSCs

**Figure 1:**
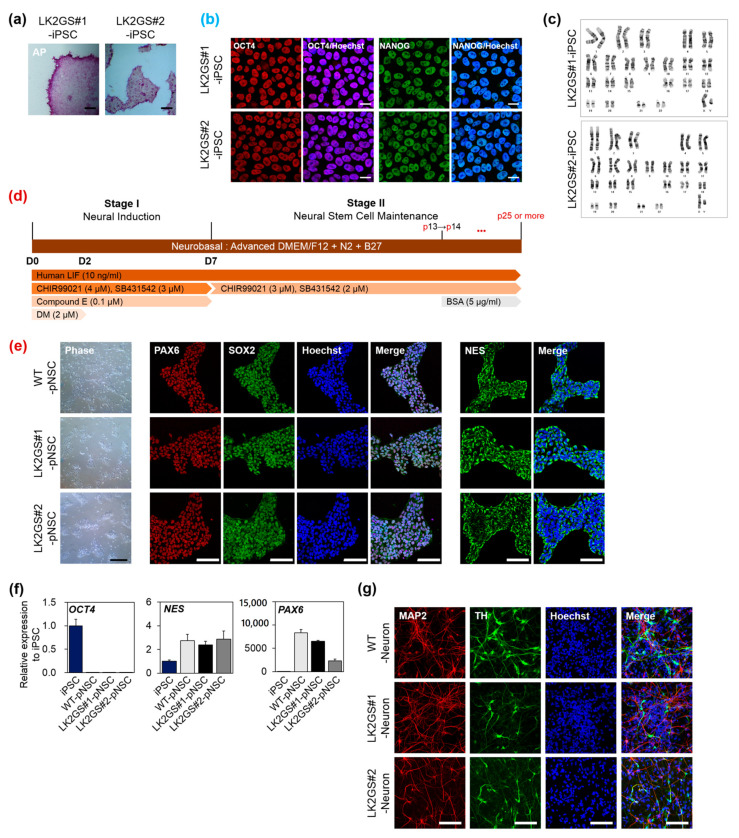
Characterization of pNSCs differentiated from PD patient-specific induced pluripotent stem cells (iPSC). (**a**) Alkaline phosphatase (AP) activity of established iPSC (Scale bar, 200 μm). (**b**) Immunostaining of established iPSC for human pluripotent stem cell markers, OCT4, and NANOG (scale bar, 50 μm). Counterstain, Hoechst33342. (**c**) Karyotypes of established iPSC. (**d**) A schematic diagram of the differentiation protocol used to obtain pNSC from iPSC. *** *p* < 0.001. (**e**) Representative morphology and immunostaining of pNSC with neural stem/progenitor markers, PAX6, SOX2, and NES (scale bar, 100 μm). (**f**) mRNA expression levels of human pluripotent stem cell marker (OCT4) and neural stem cell markers (NES and PAX6) in differentiated pNSCs. (**g**) Representative fluorescence image of differentiated neurons with neuronal cell marker, MAP2, and dopaminergic neuron marker, TH (scale bar 100 μm). LK2GS; PD patient-derived cells harboring the Gly2019Ser mutations in the leucine-rich repeat kinase 2 gene, WT; healthy control cells.

The iPSCs were differentiated into pNSCs as previously described [18] with some modifications. To start the differentiation, iPSCs, which were cultured in TeSR^TM^-E8^TM^ (STEMCELL Technologies, Vancouver, BC, Canada) were seeded on Geltrex-coated dishes at about 20% confluence with ReLeSR^TM^ (STEMCELL Technologies, Vancouver, BC, Canada). Next, 10 μM Y-27632 (Tocris, Bristol, UK) was added to the culture medium for only one day of seeding. TeSR^TM^-E8^TM^ was then switched to Neural Induction Medium (NIM: 50% Advanced DMEM/F-12, 50% Neurobasal^TM^ Medium, N-2 supplement (100×), B-27 supplement (50×) minus vitamin A, Glutamax (Thermo Fisher Scientific, Waltham, MA, USA), 10 ng/mL human LIF (Peprotech, Rocky Hill, NJ, USA), 4 μM CHIR99021 (Tocris, Bristol, UK), 3 μM SB431542 (Tocris, Bristol, UK), and 0.1 μM Compound E (Millipore, Burlington, MA, USA). Dorsomorphin (2 μM; Sigma-Aldrich, St Louis, MO, USA) was added for two days and excluded for another five days. On day 7 of differentiation, the cells were re-plated on a Geltrex-coated dish at a density of 400,000 cells/35 mm, using the Accutase^TM^ solution (Millipore, Burlington, MA, USA) with Neural Stem Cell Maintenance Medium (NSMM: 50% Advanced DMEM/F-12, 50% Neurobasal^TM^ Medium, N-2 supplement (100×), B-27 supplement (50×), minus vitamin A, Glutamax, and 10 ng/mL human LIF, 3 μM CHIR99021, 2 μM SB431542) containing 10 μM Y-27632. The pNSCs were then passaged every week using the Accutase^TM^ solution. After passage 14, cells were cultured in NSMM supplemented with 5 μg/mL BSA (Sigma-Aldrich, St Louis, MO, USA). The differentiation proceeded as Figure 1d. Differentiation was evaluated according to the immunofluorescence results using antibodies to PAX6 and SOX2, which are thought to represent characteristics of neural stem cells. Consistent with the previous report [21], the expression of either marker was confirmed in most cells after passage 4 when it was regarded as a successful differentiation to pNSCs. For proteomic analysis, pNSCs from passage 17 were used.

### 2.3. Differentiation of pNSCs into Neuronal Cells

To differentiate pNSCs into neuronal cells, pNSCs were seeded onto poly L-ornithine/laminin-coated dishes in NSMM supplemented with 5 μg/mL BSA. The next day, the medium was replaced with Neuronal differentiation medium (NDM: Neurobasal^TM^ medium supplemented with B-27 supplement (50X), minus vitamin A, 2 mM Glutamax, 20 ng/mL BDNF, 20 ng/mL GDNF, 0.5 mM dbcAMP, and 200 μM ascorbic acid. The medium was replaced every other day for 18 d.

### 2.4. RNA Extraction and Quantitative Real-Time PCR (qRT-PCR)

Total RNA was extracted from cell pellets using an RNeasy Plus Mini Kit (QIAGEN, Hilden, Germany) according to the manufacturer’s recommendations [19]. The first-strand cDNA was produced from 1 μg total RNA using the iScript™ cDNA Synthesis Kit (Bio-rad, Hercules, CA, USA) and used for performing qRT-PCR using a 7500 Fast Real-Time PCR System (Applied Biosystems, Foster City, CA, USA). The primers used in this study are listed in Supplementary Table S2.

### 2.5. Alkaline Phosphatase (AP) Staining

Leukocyte Alkaline Phosphatase Kit (Sigma-Aldrich, St Louis, MO, USA) was used according to the manufacturer’s instructions [20]. The cells were fixed in diluted 4% formaldehyde solution (Sigma-Aldrich, St Louis, MO, USA) in Dulbecco’s phosphate-buffered saline (DPBS, Welgene, Kyungsan, Korea) for 30 s, followed by two washes with Tris Buffered Saline with Tween 20 (TBST) (LPS solution, Daejeon, Korea). Then, the fixed samples were incubated in a mixture of naphthol AS-BI alkaline solution with fast red violet LB for 30 min in the dark.

### 2.6. Immunocytochemistry

The cells were fixed in diluted 4% formaldehyde solution (Sigma-Aldrich, St Louis, MO, USA) in Dulbecco’s phosphate-buffered saline (DPBS, Welgene, Kyungsan, Korea) for 10 min, followed by two to three washes with DPBS. The cells were blocked and permeabilized with 3% bovine serum albumin (BSA, Thermo Fisher Scientific, Waltham, MA, USA) and 0.3% TritonX-100 (Sigma-Aldrich, St Louis, MO, USA) in DPBS for an hour at room temperature. The cells were incubated with primary antibodies (Supplementary Table S3) in 1% BSA overnight at 4 °C, followed by two to three washes with 0.1% BSA in DPBS. Then, the cells were incubated with the matched secondary antibodies (Thermo Fisher Scientific, Waltham, MA, USA) in 0.1% BSA for an hour at room temperature. Hoechst33342 (Thermo Fisher Scientific, Waltham, MA, USA) was added to show the nuclei. Stained cells were observed with an Axiovert 200M microscope (Carl Zeiss, Oberkochen, Germany) or LSM 800 confocal microscope (Zeiss, Munich, Germany).

### 2.7. Flow Cytometry

The cells were dissociated into single cells using the Accutase^TM^ solution. For apoptosis assays, the cells were suspended in 1× Binding Buffer (BD Pharmingen^TM^, San Diego, CA, USA). Then, the cells were treated with FITC-conjugated annexin V in an Apoptosis Detection Kit and 7-AAD (BD Pharmingen^TM^, San Diego, CA, USA) for 25 min at room temperature. The cells were analyzed using a BD Accuri^TM^ C6 Flow cytometer (BD Biosciences, San Jose, CA, USA). The data were evaluated using the BD Accuri^TM^ C6 software. For the detection of superoxide and reactive oxygen species in iPSCs and iPSC-induced pNSCs, cells were treated with MitoSOX™ Red Mitochondrial Superoxide Indicator (Thermo Fisher Scientific, Waltham, MA, USA) for 10 min at 37 °C. pNSCs were then analyzed using a BD FACSVerse^TM^ Flow cytometer (BD Biosciences, San Jose, CA, USA). The data were evaluated using the FlowJo software (ver. 10).

### 2.8. Karyotyping

Karyotyping of established iPSCs was performed by GenDix (Seoul, Korea) according to the manufacturer’s instructions [22].

### 2.9. In-Solution Tryptic Digestion

The cells were lysed and mixed with rehydration solution (7 M urea, 2 M thiourea, 4% HAPS). The protein solution was reduced by 5 mM DTT and then incubated with 14 mM iodoacetamide for alkylation of the reduced proteins. Excess iodoacetamide was quenched by adding DTT to a final concentration of 7 mM. The reduced and alkylated proteins were digested by treatment with trypsin (2% *w*/*w*) at 37 °C overnight. This reaction was terminated by the addition of 2% formic acid. The tryptic peptides were dried in a speed-vac.

### 2.10. LC-MS/MS Analysis and Database Search

The resulting trypsin digested peptides were separated and analyzed using reversed-phase capillary high-performance liquid chromatography directly coupled to a Thermo LTQ Orbitrap mass spectrometer following the procedure described [23], with slight changes. Briefly, both a 0.075 × 20 mm trapping column and a 0.075 × 120 mm resolving column were packed with C18AQ 218MS low formic acid C18 beads (5 μm in size, 200Å pore size; C18AQ, Michrom BioResources, Auburn, CA, USA) and placed in-line. Peptides were bound to the trapping column for 10 min with 2% (vol/vol) aqueous cetonitrile containing 0.1% (vol/vol) formic acid. The peptides were eluted with a gradient of 2% to 90% (vol/vol) acetonitrile containing 0.1% (vol/vol) formic acid at a flow rate of 0.2 μL/min. For tandem mass spectrometry, full mass scan range mode was set at *m*/*z* = 50 to 2000 Da. After determining the charge states of the ion zoom scans, product ion spectra were acquired in MS/MS mode with relative collision energy of 55%. Individual spectrum from MS/MS was processed using Protein discoverer 2.1 software (Thermo scientific, Waltham, MA, USA). Processed ions were sequenced and mapped against the Swiss-Prot database using the MASCOT DAEMON program (http://www.matrixscience.com). Peptides were restricted to trypsin fragments with a maximum of one missed cleavage, cysteine carbamidomethylation, and identification of the protein in at least two out of three replicate injections.

### 2.11. Western Blotting

The cells were washed with DPBS two to three times and harvested with ice-cold protein lysis buffer (1% [*v*/*v*] Triton X-100, 5 mM ethylenediaminetetraacetic acid (EDTA, Thermo Fisher Scientific, Waltham, MA, USA), 1 mM phenylmethanesulfonyl fluoride (PMSF, Thermo Fisher Scientific, Waltham, MA, USA), 100× Xpert Protease Inhibitor Cocktail Solution (GenDEPOT, Katy, TX, USA), and PhosSTOP^TM^ phosphatase inhibitor cocktail tablets (Roche, Basel, Switzerland). Protein quantification was performed using a Protein Assay Dye Reagent Concentrate (Bio-rad, Hercules, CA, USA). Equal amounts of total protein were boiled for 5 min and loaded for SDS-PAGE. The proteins were transferred onto a PVDF membrane (Bio-rad, Hercules, CA, USA) using a Wet/Tank Blotting Systems (Bio-rad, Hercules, CA, USA). The membranes were incubated with 5% blocking solution (Difco^TM^ Skim milk, BD Biosciences) in TBST (0.05% Tween^®^ 20 (Sigma-Aldrich, St Louis, MO, USA) in TBS (LPS solution, Daejeon, Korea), and then incubated with primary antibodies (Supplementary Table S3) overnight at 4 °C. The membranes were then incubated with horseradish peroxidase (HRP)-conjugated secondary antibodies (Cell Signaling Technology, CST, Danvers, MA, USA). For detection of the signals from HRP, we added ECL^TM^ Select Western Blotting Detection Reagent (GE Healthcare, Chicago, IL, USA). Images of protein bands were acquired using a LAS-3000 imaging system (Fujifilm, Minato, Tokyo, Japan).

### 2.12. Statistical Analysis

Statistical analysis was performed using GraphPad Prism V5 (GraphPad Software, Inc., San Diego, CA, USA). All data are presented as means ± standard error of means (SEM), and the statistical results were analyzed by unpaired, two-tailed Student’s *t*-test. *p*-values less than 0.05 were considered statistically significant.

## 3. Results

### 3.1. Generation and Characterization of pNSCs from PD Patients Cells Harboring LRRK2 G2019S

In previous study, we established iPSCs from somatic cells of PD patients harboring the *LRRK2 G2019S* mutation (LK2GS#1-, LK2GS#2-iPSCs), using non-integrating oriP/EBNA-1-based episomal vectors [19]. We analyzed pluripotency by immunostaining for octamer-binding transcription factor 4 (OCT4) and NANOG as well as alkaline phosphatase reactivity (Figure 1a,b). It was confirmed that OCT4 and NANOG were expressed in almost all iPSCs and located in the nucleus (Figure S1a).

We also confirmed the normal karyotype as a result of the genomic stability analysis of LK2GS#1- and LK2GS#2-iPSC (Figure 1c). To generate pNSCs, we differentiated patient-iPSCs through a protocol which consisted of two stages, neural induction (Stage 1) and pNSC maintenance (Stage 2) (Figure 1d) [18]. For the characterization of differentiated pNSCs, we performed immunostaining and qRT-PCR for diverse neural stem cell markers. As shown in Figure 1e, two types of LK2GS-pNSCs from iPSCs (LK2GS#1-pNSC and LK2GS#1-pNSC) and WT-pNSC prominently expressed neural stem cell markers, including paired box 6 (PAX6), SRY-Box transcription factor 2 (SOX2), and Nestin (NES). The qRT-PCR analysis showed that pNSCs expressed endogenous neural stem/progenitor markers, Nestin (*NES*), and *PAX6*. In contrast, the expression of undifferentiated stem cell pluripotency marker, *OCT4*, was dramatically repressed in the pNSCs, suggesting that pNSCs are fully differentiated into neural lineage (Figure 1f). Furthermore, we performed the spontaneous differentiation of pNSCs to verify the differentiation potential. As shown in Figure 1g, it was confirmed that microtubule-associated protein 2 (MAP2)-positive neurons containing tyrosine hydroxylase (TH) appeared in these differentiated cells, thereby confirming the differentiation potential of pNSCs. We found there were no differences in differentiated neurons between WT- and LK2GS-pNSCs in terms of the expression of TH-positive cells (Figure S1b,c). These data indicated the successful generation of both LK2GS-iPSCs and WT-iPSC into differentiated pNSCs.

### 3.2. Functional Characerization for Apoptosis and Oxidative Stress in pNSCs

**Figure 2:**
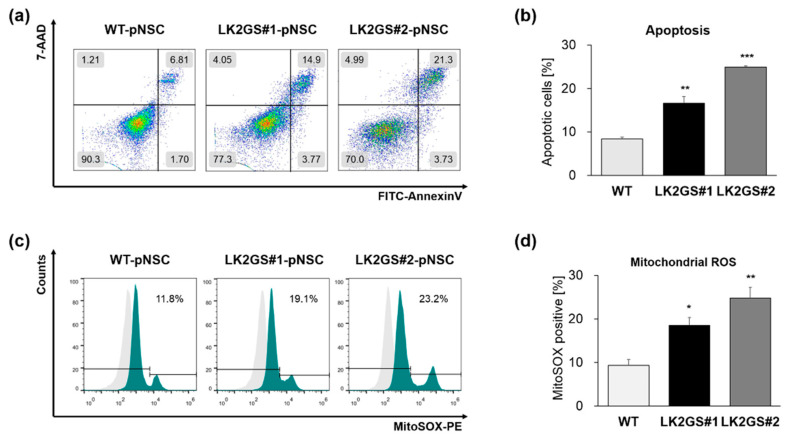
Functional characterization of LK2GS-pNSCs. (**a**) Detection of apoptotic cells by annexin V and 7-AAD staining. (**b**) Bar graph indicating the averages of annexin V positive populations. (**c**) Detection of mitochondrial reactive oxygen species (ROS) by the detection reagent MitoSOX Red. (**d**) Bar graph indicating the averages of MitoSOX Red positive populations. Results are means ± standard error of mean (S.E.M) for three independent experiments. * *p* < 0.05, ** *p* < 0.01, *** *p* < 0.001.

Pathogenic mutations of the GTPase or the kinase domain in the LRRK2 gene are associated with PD through the increased kinase activity, which affects various cellular functions, including mitochondrial regulation [24,25,26]. Accordingly, we confirmed that LRRK2 is expressed in pNSCs and the kinase activities of LRRK2, which have been investigated by the expression of phosphorylated-LRRK2 at Ser935 [27], were significantly increased in LK2GS-pNSCs (LK2GS#1-pNSC, LK2GS#2-pNSC) in comparison with WT-pNSC (Figure S2). It is already well known that LRRK2 mutants are involved in apoptosis and oxidative stress [28]. Therefore, we next investigated whether there are more apoptotic cells in LK2GS-pNSC, compared with WT-pNSC. We compared the apoptotic cells in LK2GS-pNSCs (LK2GS#1-pNSC, LK2GS#2-pNSC) and WT-pNSC using annexin V/7-AAD analysis (Figure 2a,b). We found that cellular apoptosis was significantly increased in LK2GS-pNSCs compared with that of WT-pNSC. It is well known that LK2GS PD patient-specific neural cells recapitulate mitochondrial defects [25]. Accordingly, we investigated the mitochondrial reactive oxygen species (ROS) production in LK2GS-pNSCs and WT-pNSC using MitoSOX superoxide indicator. As expected, MitoSOX-positive cells are significantly increased in LK2GS-pNSCs compared with that of WT-pNSC (Figure 2c,d). These results suggest that LK2GS-pNSCs undergo oxidative-stress-mediated cellular apoptosis, compared to WT-pNSC.

### 3.3. Ontological Classification of Differentially Regulated Proteins Between WT and LK2GS-pNSC

**Figure 3:**
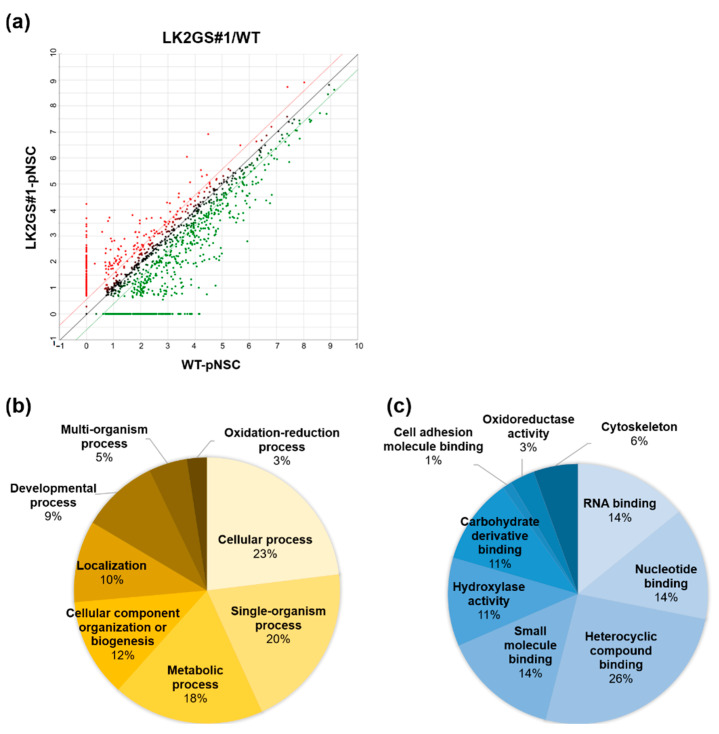
Ontology classification of differentially expressed proteins (DEPs) in the proteomics analysis. (**a**) Scatter plot of proteomics data of WT-pNSC vs LK2GS#1-pNSC. Functional classification of the identified proteins according to their (**b**) biological processes and (**c**) molecular functions by DAVID bioinformatics resources.

To determine the difference in proteome profiling between WT-pNSC and LK2GS-pNSC, we conducted LC-MS/MS using extracted whole proteins. A scatter plot of proteome data of WT-pNSC vs. LK2GS#1-pNSC is shown in Figure 3a. Upregulated proteins in LK2GS#1-pNSC are red, and down-regulated proteins in LK2GS#1-pNSC are green. We identified a total of 1452 proteins, of which 913 had fold changes of 1.5 or more against score in LK2GS#1-pNSC compared with that of WT-pNSC. We listed the top 10 proteins with the most changes between WT-pNSC and LK2GS#1-pNSC in Table S4. To characterize the DEPs, 913 proteins were categorized by functional involvement as documented in the DAVID Gene Ontology database (http://david.abcc.ncifcrf.gov) and UniProt (http://www.uniprot.org) websites, and the results are represented graphically. DEPs were assigned to eight biological processes (Figure 3b) and nine molecular functional categories (Figure 3c) according to the database search and function exploration. The eight biological processes were cellular process (23%), single organism process (20%), metabolic process (18%), cellular component organization or biogenesis (12%), localization (10%), developmental process (9%), multi-organism process (5%), and oxidation-reduction process (3%). Additionally, based on the molecular function headings, the proteins were predicted to be associated with heterocyclic compound binding (26%), RNA binding (14%), nucleotide-binding (14%), small molecule binding (14%), hydrolase activity (11%), carbohydrate derivative binding (11%), cytoskeleton molecule (6%), oxidoreductase activity (3%), and cell adhesion molecule binding (1%) (Figure 3c). Among the various these functions, we noted that PD is closely related to oxidoreductase activity, cell adhesion, and cytoskeleton. We listed proteins related to oxidoreductase activity, cell adhesion, and cytoskeleton in Table 1.

### 3.4. Regulation of Oxidative Stress, Cell Adhesion, Cytoskeleton-Related Proteins in LK2GS-pNSCs

**Figure 4:**
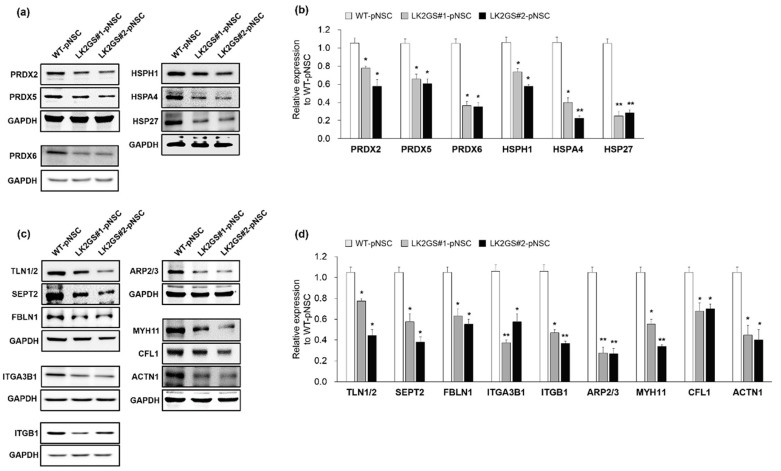
Confirmation of DEPs related to PD pathologies. (**a**) The same amounts of total proteins prepared from WT, LK2GS#1- and LK2GS#2-pNSC were subjected to Western blot analysis with oxidative stress-related antibodies including PRDX2, PRDX5, PRDX6, HSPH1, HSPA4, and HSP27. GAPDH was employed as a loading control. (**b**) Quantification of protein expression levels after normalization using GAPDH. (**c**) Western blot confirmation of the cell adhesion, ECM related proteins, including TLN1/2, CFL1, FBLN1, ITGA3B1, ITGB1 and ARP2/3, and cytoskeleton molecules, including MYH11, SETP2, and ACTN-1. GAPDH was employed as a loading control. (**d**) Histograms showing the fold changes of their relative expression levels. Results are means ± S.E.M for three independent experiments. * *p* < 0.05, ** *p* < 0.01, compared with WT-pNSC.

It is well known that oxidative stress, which is involved in oxidoreductase activity and the oxidation-reduction process, is one of the most important factors in several types of degenerative diseases [29,30,31,32]. Cell adhesion molecules also play key roles in PD development [33,34,35], and cell adhesion processes are mediated by the binding of ECM proteins, which are activated by cytoskeletal components [36]. In addition, cytoskeleton-related proteins are actin filament binding and development regulating proteins that play crucial roles in cell division, cell motility, and cell migration [37,38]. Consistently, we found that oxidoreductase activity-, cell adhesion-, and cytoskeleton-related molecules were differentially expressed in the LK2GS-pNSC with respect to their molecular functions. Therefore, we validated the molecules mentioned above through Western blotting (Figure 4 and Table 1). In this confirmation, we included protein samples obtained from LK2GS#2-pNSC to verify whether both LK2GS#1- and LK2GS#2-pNSCs gave rise to similar results. Regarding oxidoreductase activity-related proteins, we investigated the expression levels of peroxiredoxin-2 (PRDX2), peroxiredoxin-5 (PRDX5), peroxiredoxin-6 (PRDX6), heat shock protein 105 (HSPH1), and heat shock 70 kDa protein 4 (HSPA4) (Figure 4a,b). We observed that PRDX2, PRDX5, PRDX6, HSPH1, and HSPA4 levels were decreased in both LK2GS#1- and LK2GS#2-pNSCs compared to WT-pNSC. We also examined the expression pattern of heat shock protein 27 (HSP27), a representative oxidative stress regulator in HSP, and found its expression pattern to be consistent with that of other HSP family proteins. Next, we verified the expression of cell adhesion-related proteins, including talin-1/2 (TLN1/2), cofilin-1 (CFL1), and several ECM-related proteins, such as fibulin-1 (FBLN1), integrin alpha-3-beta-1 (ITGA3B1), integrin beta-1 (ITGB1), and actin-related proteins-2/3 (ARP2/3) between WT- and LK2GS-pNSCs (Figure 4c,d). The expression levels of cell adhesion (TLN1/2 and CFL1) and several ECM-related proteins (FBLN1, ITGA3B1, ITGB1, and ARP2/3) were significantly increased in WT-pNSC compared to the decreased expression levels observed in both LK2GS-pNSCs. Lastly, we evaluated specific proteins of interest that may play potential roles as cytoskeleton molecules, including myosin heavy chain 11 (MYH11), septin-2 (SEPT2), and actinin alpha 1 (ACTN1) (Figure 4c,d). We confirmed that the expression patterns of MYH11, SEPT2, and ACTN1 were significantly decreased in both LK2GS#1- and LK2GS#2-pNSC compared to WT-pNSC. Taken together, the LK2GS-pNSCs is a reliable in vitro experimental model that reflects expression changes related to PD in protein expression profiles.

### 3.5. Induction of DNA Damage-Mediated Apoptosis in LK2GS-pNSCs

**Figure 5:**
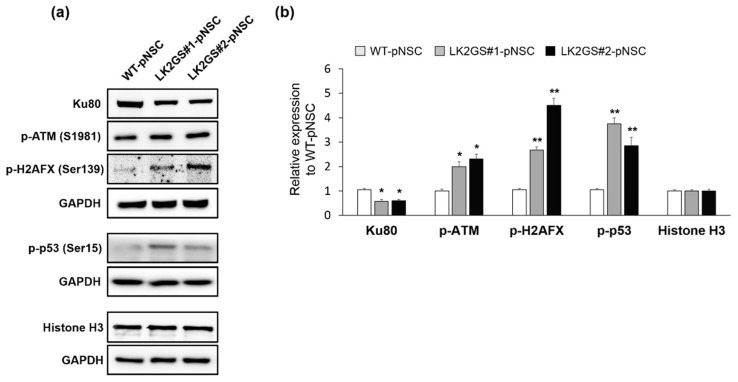
Expression of double stranded break-associated proteins in WT and LK2GS-pNSCs. (**a**) Western blots showing the expression of key components involved in the double-stranded break, such as Ku80, p-ATM, p-H2AFX, and p-p53. GAPDH and histone H3 were loading controls. (**b**) Histograms showing the fold changes of their relative expression levels. The data shown are the means ± S.E.M of three independent experiments. * *p* < 0.05, ** *p* < 0.01, compared with WT-pNSC.

We then investigated whether the apoptotic cell death in LK2GS-pNSCs was affected by the DNA damage response. Among the specifically downregulated proteins in LK2GS#1-pNSC compared with WT-pNSC, we identified Ku80 (also known as X-ray repair cross-complementing 5, XRCC5), a well-known DNA damage repair protein. That consists of Ku70/Ku80 heterodimers that participate in the non-homologous end-joining (NHEJ) pathway of DNA repair [39]. Ataxia-telangiectasia mutated (ATM) kinase activity is activated under oxidative stress and plays an important role in DNA-damage-mediated signaling [40], and is also regulated by Ku80 [41,42]. Based on these references, we speculated that, under the oxidative stress, reduced Ku80 might upregulate the ATM kinase activity, and then the apoptotic signal is transmitted by ATM kinase activation to induce apoptotic changes in the cells. With this hypothesis, we examined the expression levels of Ku80 and the status of phosphorylation of ATM on Ser1981 (S1981) that normally occurs in response to oxidative stress, critical for sustained occupancy of ATM on DNA DSB sites [40,43]. Our results revealed that Ku80 was dramatically downregulated in both LK2GS#1- and LK2GS#2-pNSC, and its expression levels were 0.58 ± 0.08- and 0.61 ± 0.05-fold lower than that of WT-pNSC. Phosphorylated ATM (p-ATM) S1981 was significantly increased in both LK2GS#1- and LK2GS#2-pNSC (Figure 5a,b). Auto-phosphorylation of ATM subsequently modifies a downstream regulator, such as tumor protein p53 (p53) Ser15, checkpoint kinase 2 (CHEK2) Thr68, mouse double minute 2 homolog (MDM2), and H2A histone family member X (H2AFX) Ser139, through its activation of the kinase in response to DSBs [44]. For these reasons, we observed the expression levels of phosphorylated H2AFX (p-H2AFX) and p53 (p-p53) in LK2GS-pNSCs compared with that of WT-pNSC (Figure 5a,b). Consistent with p-ATM (S1981), p-H2AFX and p-p53 were also upregulated in LK2GS-pNSCs. Taken together, these results suggested that cellular oxidative stress in LK2GS-pNSCs might induce DNA damage, followed by an activation of consecutive ATM-mediated signaling (i.e., phosphorylation of p53 and H2AFX) via its kinase activity.

## 4. Discussion

PD is characterized by the degeneration of midbrain dopamine neurons, ultimately leading to progressive movement disorder in patients. Pathogenic mutations of several genes, such as *LRRK2*, Parkinsonism Associated Deglycase (*PARK7)*, PTEN-induced kinase 1 (*PINK1)*, Parkin RBR E3 Ubiquitin Protein Ligase (*PRKN)*, and Alpha-synuclein (*SNCA)*, are known to induce PD [45]. Several factors, including mitochondrial dysfunction, autophagy, and oxidative stress, are also known to be involved in the pathogenesis of PD [46]. In this study, we performed comparative proteomic analysis between pNSCs from PD patient harboring the *LRRK2 G2019S* mutation and healthy control, in an attempt to identify and characterize the DEPs (Figure 3). LK2GS#1-pNSC, which was used to the proteomic analysis, showed increased level of mitochondrial ROS, which may act as one of the major factors involved in PD pathogenesis [47,48]. In confirmation of the proteomic results, several oxidative stress response proteins, such as PRDX2, PRDX5, and PRDX6, were downregulated in LK2GS-pNSCs relative to the WT-pNSC (Figure 4a). PRDX2, PRDX5, and PRDX6 are glutathione peroxidases; therefore, they are believed to play an important role in eliminating the H_2_O_2_ generated as a byproduct of metabolism in the cytosol. ROS are degraded by peroxiredoxin (Prx), glutathione peroxidase (GPx), and catalase (CAT), which convert H_2_O_2_ to O_2_ and H_2_O [49]. Exogenous and endogenous oxidative stress generates ROS, resulting in cellular injury, necrosis, and apoptosis [50]. In addition, we also identified and classified HSPs (HSPH1, HSPA4, and HSP27) as DEPs between WT-pNSC and LK2GS-pNSCs (Figure 4a). Their chaperon and antioxidant functions are correlated with their apoptosis-regulating functions against stress. HSPs were originally discovered based on their induction in high temperature conditions. They primarily act as molecular chaperons facilitating the folding of other cellular proteins, but also regulating apoptosis by interacting directly with key components of the apoptotic pathway [51]. Hence, we speculated that the downregulation of these molecules is related to the increased level of ROS, which may be related to the LK2GS-pNSCs.

Moreover, we found that the protein expression levels of TLN1/2, CFL1, FBLN1, ITGA3B1, ITGB1, and ARP2/3 were down-regulated in LK2GS-pNSCs relative to the WT-pNSC (Figure 4b). In our proteomic analysis, we identified and focused on TLN1/2 and CFL1 in cell adhesion molecule binding categories because they are known to be regulated by LRRK2 [52,53]. CFL1 plays a key role as an actin depolymerizing factor in actin dynamics, which are implicated in neuronal function [54]. TLN is a large multi-domain cytosolic protein that binds to the cytoplasmic domain of integrin β subunits. They activate integrins and couple them to the actin cytoskeleton, and regulate integrin signaling [55]. In addition, cell adhesion molecules are crucial for the assembly of individual cells into the three-dimensional tissues of animals. Many cell adhesion mechanisms are also responsible for assembling cells and, along with their connections to the internal cytoskeleton, determine the overall architecture of the tissue. In this regard, cell adhesion molecules are typically multi-protein complexes composed of three general classes of proteins: cell adhesion molecules/adhesion receptors, ECM proteins, and cytoplasmic plaque/peripheral membrane proteins [33,56,57]. For these reasons, we also analyzed the expression levels of ECM-related proteins, including FBLN1, ITGA3B1, ITGB1, and ARP2/3, which were downregulated in LK2GS-pNSCs. Integrins are heterodimeric cell-surface molecules that regulate the production of second messengers within the cells to provide a direct link between the ECM and cytoskeleton. In addition, integrins are involved in organogenesis, anchoring of stem cells to niches, regulation of gene expression, cell proliferation, differentiation, migration, and death [58,59]. As integrin dysfunction is involved in the pathogenesis of several disease states, many studies have targeted them for studying neurodegenerative diseases [34,35]. FBLN-1 is an ECM protein as well as an intracellular integrin β1-binding protein that plays an important role in the structure of elastic fibers and basement membranes of various tissues. During development, FBLN-1 is prominently expressed in neural crest cells, implicated in the morphogenesis of the cranial neural crest-derived structures [60,61]. The ARP2/3 complex is a stable multiprotein complex composed of seven subunits, two of which are structurally related to actin. In the cell, ARP2/3 generates branched actin networks when activated by nucleation-promoting factors (NPFs). The pushing force induced by branched actin networks is critical for lamellipodial protrusions [62]. Among the protein categories investigated, cytoskeleton-related molecules, including MYH11, SEPT2, and ACTN1 were highly downregulated in LK2GS-pNSCs compared with that of WT-pNSC. The cytoskeleton is composed of actin and microtubule filaments. Actin is a major cytoskeleton protein in neurons, and the dynamics of their assembly are involved in a variety of aspects of cell motility, vesicle transport, and membrane turnover [63,64,65]. Thus, the disorganization of actin results in failure of cell differentiation, cell migration, cell mobilization, and engraftment [37,66].

Interestingly, we also found that Ku80 is significantly downregulated in LK2GS-pNSCs (Table 1 and Figure 5). Ku80 is a highly abundant protein found in vivo as a stable heterodimer consisting of two subunits, Ku70 and Ku80, associated with the NHEJ pathway of DNA DSB repair. Because neurons are usually regarded as post-mitotic cells, NHEJ is the primary pathway of DSB repair in neurons [39]. DNA DSBs are recognized by the Ku70/Ku80 heterodimer, which binds the DNA ends and then recruits and activates the catalytic subunit of the DNA-PKcs (DNA-dependent protein kinase, catalytic subunit) in NHEJ. Then, activation of DNA-PKcs allows end-processing activities such as the ARTEMIS, APLF (nucleases), and PNKP (polynucleotide kinase/phosphatase) to access the broken DNA ends and prepare them for ligation [39]. Recently, it has been shown that Ku70/Ku80 regulates the ATM signaling pathway [42], which is involved in DNA-damage-related apoptosis [40]. When DNA is damaged by genotoxic stress such as ROS by UV, ATM phosphorylates the tumor suppressor p53 [44]. Subsequently, phosphorylated p53 induces its target gene via phosphorylation, followed by the activation of caspases [67]. In this study, Ku80 was significantly downregulated in LK2GS#1- and LK2GS#2-pNSCs, which triggers the induction of the DNA damage-repair protein ATM phosphorylation, followed by activation of the p53 pathway.

In summary, we carried out a comparative proteomic analysis and identified the differentially expressed protein of LK2GS-NSC compared with WT-NSC. Since pathogenic features of PD only become apparent at certain ages and only in specific brain region, the dopaminergic neurons of the substantia nigra pars compacta, it is striking that significant changes are detected in LK2GS-pNSCs at the proteomic level. Furthermore, we found that LK2GS-pNSCs were highly susceptible to oxidative stress, thereby increasing apoptotic cell death. Through proteomic analysis and validation, LK2GS-pNSCs also exhibited the dysregulation of the oxidative stress, ECM and cell adhesion-associated proteins, which affected the cytoskeleton-associated proteins. Further studies are needed to confirm that these molecules function directly in the pathogenesis of PD and the LRRK2-associated pathway. In addition, previous studies have shown that the incidence and prevalence of PD are different between men and women [68,69,70]. Therefore, it is necessary to investigate whether there is reproducibility in samples derived from a larger number of PD patients compared to age- and gender-matched healthy controls. To our knowledge, our findings provide the first report on the quantitative proteomic analysis of pNSCs differentiated from *LRRK2 G2019S* PD patient-derived iPSCs. Considering the increasing interests in disease-specific pNSCs, our results contain useful information and insights about the pathogenesis and neurodegeneration mechanisms of PD.

## Figures and Tables

**Table 1:** Identification of differentially expressed protein in WT-pNSC and LK2GS-pNSC.

Acession no.	Protein Name	MW (kDa)	*pI* * Value	Coverage (%) ^†^	Matched Peptides	Score		Fold Change (LK2GS/WT)
						WT	LK2GS	
Oxidoreducates activity								
P32119	Preoxiredoxin-2 (PRDX2)	21.9	5.97	34.34	11	73.3	48.2	0.66
P30044	Peroxiredoxin-5 (PRDX5)	22.1	8.7	25.7	3	8.38	3.92	0.47
P30041	Peroxiredoxin-6 (PRDX6)	25	6.38	23.66	3	18.55	5.8	0.31
Q92598	Heat shock protein 105 (HSPH1)	96.8	5.39	12.24	7	13.13	6.5	0.5
P34932	Heat shock 70 kDa protein 4 (HSPA4)	94.3	5.19	12.98	7	24.6	14.56	0.6
Q9BRX8	Redox-regulatory protein FAM213A (F213A)	25.7	8.84	6.55	1	4.65	0	WT unique
Q9NRD8	Dual oxidase 2 (DUOX2)	175.3	7.85	1.03	1	0	3.4	LK2GS unique
Q6DKJ4	Nucleoredoxin (NXN)	48.4	4.97	3.68	1	2.33	0	WT unique
P21266	Glutathione S-transferase Mu 3 (GSTM3)	26.5	5.54	7.65	1	2.99	0	WT unique
P10599	Thioredoxin (TXN)	11.7	4.9	8.57	1	1.71	0	WT unique
Q96SL4	Glutathione peroxidase 7 (GPX7)	21	8.27	15.51	1	0	2.3	LK2GS unique
Q8N543	2-oxoglutarate and iron-dependent oxygenase domain-containing protein 1 (OGFD1)	63.2	5.11	4.06	1	2.41	0	WT unique
Q9BUT1	3-hydroxybutyrate dehydrogenase type 2 (BDH2)	26.7	7.65	19.59	3	4.1	6.3	1.5
Q9C0B1	Alpha-ketoglutarate-dependent dioxygenase FTO (FTO)	58.2	5.22	6.35	2	10.3	3.9	0.37
P31150	Rab GDP dissociation inhibitor alpha (GDIA)	50.6	5.14	21.03	8	88.02	35.94	0.41
P50395	Rab GDP dissociation inhibitor beta (GDIB)	50.6	6.47	39.1	15	130.3	61.6	0.47
Q15738	Sterol-4-alpha-carboxylate 3-dehydrogenase, decarboxylating (NSDHL)	41.9	8.06	6.43	1	2.6	0	WT unique
Q8N183	Mimitin, mitochondrial (MIMIT)	19.8	8.98	3.55	1	1.93	0	WT unique
P28331	NADH-ubiquinone oxidoreductase 75 kDa subunit, mitochondrial (NDUS1)	79.4	6.23	20.2	1	2.16	0	WT unique
O60701	UDP-glucose 6-dehydrogenas (UGDH)	55	7.12	14.57	4	4.65	2.71	0.58
P20674	Cytochrome c oxidase subunit 5A, mitochondrial (COX5A)	16.8	6.79	31.33	2	17.12	6.6	0.39
Q9UDR5	Alpha-aminoadipic semialdehyde synthase, mitochondrial (AASS)	102.1	6.61	1.94	1	5.86	1.94	0.33
P43304	Glycerol-3-phosphate dehydrogenase, mitochondrial (GPDM)	80.8	7.69	4.26	1	2.33	0	WT unique
P40926	Malate dehydrogenase, mitochondrial (MDHM)	35.5	8.69	35.8	9	46.65	11.41	0.24
Cell adhesion molecule binding								
Q9Y490	Talin-1 (TLN1)	269.6	6.07	5.51	5	26.58	14.37	0.54
Q9Y4G6	Talin-2 (TLN2)	271.4	5.57	1.06	1	2.24	0	WT unique
P23528	Cofilin-1 (CFL1)	18.5	8.09	44.58	6	95.82	46.26	0.48
Q92616	Translational activator GCN1 (GCN1L)	292.6	7.46	2.28	3	10.01	3.84	0.38
P04075	Fructose-bisphosphate aldolase A (ALDOA)	39.4	8.09	26.1	8	39.43	15.43	0.39
P06733	Alpha-enolase (ENOA)	434	7.39	48.62	17	232.4	106.1	0.47
P26038	Moesin (MOES)	67.8	6.4	13	8	45	22.64	0.5
Q13813	Spectrin alpha chain, non-erythrocytic 1 (SPTN1)	284.4	5.35	1.94	3	1.53	0	WT unique
Cytoskeleton molecules								
P35749	Myosin-11 (MYH11)	227.2	5.5	1.67	3	6.65	1.81	0.27
Q15019	Septin-2 (SEPT2)	41.5	6.6	20.78	4	20.12	7.3	0.36
P12814	Alpha-actinin-1 (ACTN1)	103	5.41	2.47	2	9.02	0	WT unique
P62873	Guanine nucleotide-binding protein G(I)/G(S)/G(T) subunit beta-1 (GBB1)	37.4	6	6.18	2	5.76	3.73	0.65
P62158	Calmodulin (CALM)	16.8	4.22	46.98	3	26.26	14.79	0.56
P37231	Peroxisome proliferator-activated receptor gamma (PPARG)	57.6	5.94	3.37	1	1.88	0	WT unique
DNA damage response								
P13010	86 kDa subunit of Ku antigen (XRCC5)	82.7	5.81	19.67	10	69.55	31.34	0.45

* The isoelectric point of the intact protein. ^†^ The percentage of matched sequence in the total protein sequence (the percentage of the database protein sequence covered by matching peptides).

## Supplementary Materials

The following are available online at https://www.mdpi.com/2075-1729/10/12/331/s1, Figure S1: Analysis of WT-pNSC- and LK2GS-pNSC-derived differentiated neurons. (a) Quantification of the percentage of TH positive cells. Total cells were quantified by Hoechst33342 staining. (b) Quantification of the percentage of TH/MAP2 cells. WT, WT-pNSC; LK2GS, LK2GS#1-pNSC and LK2GS#2-pNSC. Data are mean ± standard error of mean (SEM). *p*-values were analyzed using the unpaired two-tailed student’s *t*-test (N.S., not significant). Figure S2: Expression of LRRK2 in pNSCs. (a) Protein expression level of p-LRRK2 and total LRRK2 in pNSCs. Actin was used as an internal control. (b) Quantification of the protein expression level of LRRK2 in pNSCs. (c) Quantification of the kinase activity of LRRK2 in pNSCs through p-LRRK2/total LRRK2 expression. Data are mean ± standard error of mean (SEM). *p*-values were analyzed using the unpaired two-tailed student’s *t*-test (* *p* < 0.05)., Figure S3: All Western blot results used for validation of proteomic analysis. The boxed blots (blue) are shown in the main figure. Table S1: List of cells used in this study, Table S2: List of primer sequences used in this study, Table S3: List of antibodies used in this study, Table S4: Lists of the 10 proteins with the highest differences between WT-pNSC and LK2GS-pNSC.
