# Supplementary material for: Quantitative Proteomic Analysis of Primitive Neural Stem Cells from LRRK2 G2019S-Associated Parkinson’s Disease Patient-Derived iPSCs
Source: Life (Basel). 2020 Dec 7;10(12):331. doi: 10.3390/life10120331 (PMC7762312; doi:10.3390/life10120331)
Supplement: Supplementary file 1 [file life-10-00331-s001.pdf]

life

MDPI

Supplementary Materials

Quantitative proteomic analysis of primitive neural stem cells from LRRK2 G2019S-associated Parkinson's disease patient-derived iPSCs

Hyuna Sim [1,2,†], Ji-Hye Seo [3,†], Jumi Kim [3], Minyoung Oh [1,2], Joo-Eun Lee [1], Areum Baek [1], Seo-Young Lee [4], Sun-Ku Chung [5], Mi-Young Son [1,2], Jung-Il Chae [3], Young-Joo Jeon [1,*], Janghwan Kim [1,2,*]

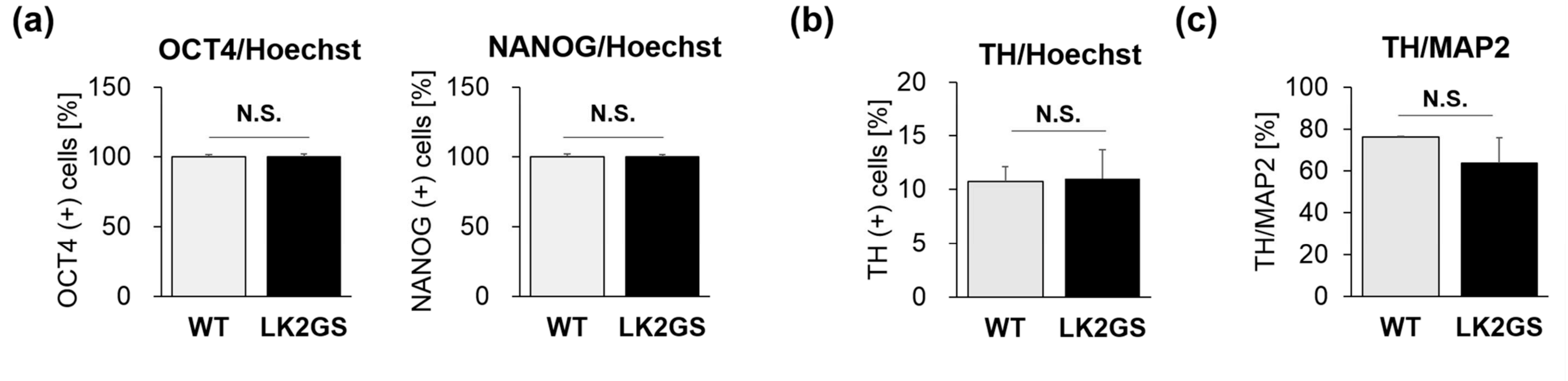

Figure S1. Analysis of WT-pNSC- and LK2GS-pNSC-derived differentiated neurons. (a) Quantification of the percentage of TH positive cells. Total cells were quantified by Hoechst33342 staining. (b) Quantification of the percentage of TH/MAP2 cells. WT, WT-pNSC; LK2GS, LK2GS#1-pNSC and LK2GS#2-pNSC. Data are mean ± standard error of mean (SEM). *p*-values were analyzed using the unpaired two-tailed student's *t*-test (N.S., not significant).

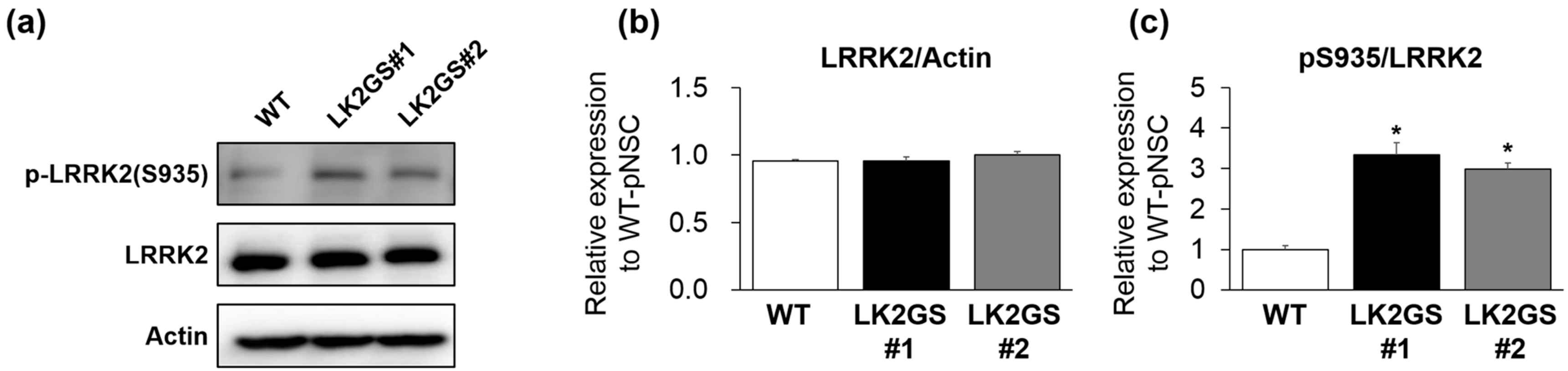

Figure S2. Expression of LRRK2 in pNSCs. (a) Protein expression level of p-LRRK2 and total LRRK2 in pNSCs. Actin was used as an internal control. (b) Quantification of the protein expression level of LRRK2 in pNSCs. (c) Quantification of the kinase activity of LRRK2 in pNSCs through p-LRRK2/total LRRK2 expression. Data are mean ± standard error of mean (SEM). *p*-values were analyzed using the unpaired two-tailed student's *t*-test (**p* < 0.05).,.

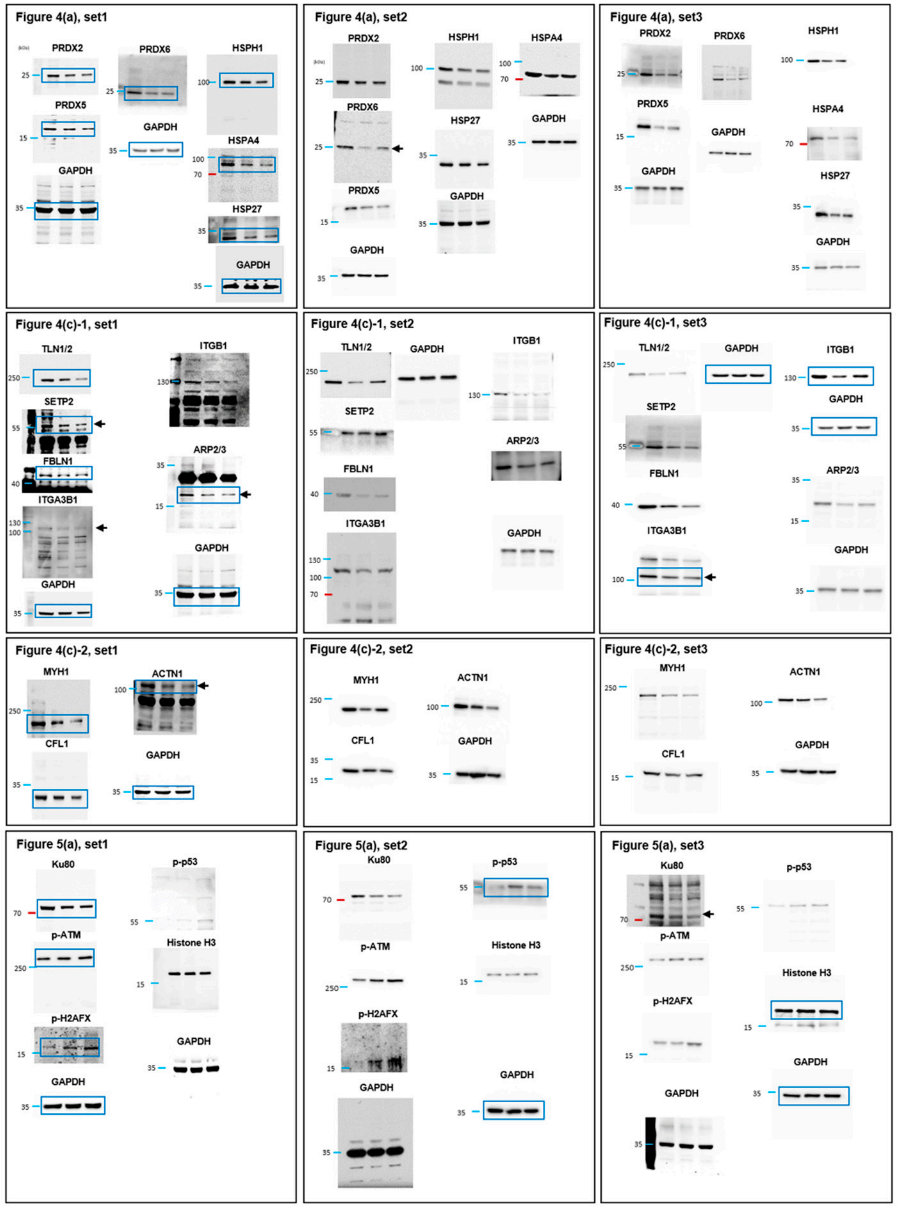
Figure 4(a), set1
PRDX2
PRDX6
HSPH1
PRDX5
GAPDH
HSPA4
GAPDH
HSP27
GAPDH
Figure 4(a), set2
PRDX2
HSPH1
HSPA4
PRDX6
HSP27
GAPDH
PRDX5
GAPDH
GAPDH
Figure 4(a), set3
PRDX2
PRDX6
HSPH1
PRDX5
GAPDH
HSPA4
GAPDH
HSP27
GAPDH
Figure 4(c)-1, set1
TLN1/2
ITGB1
SETP2
FBLN1
ARP2/3
ITGA3B1
GAPDH
GAPDH
Figure 4(c)-1, set2
TLN1/2
GAPDH
ITGB1
SETP2
ARP2/3
FBLN1
ITGA3B1
GAPDH
Figure 4(c)-1, set3
TLN1/2
GAPDH
ITGB1
GAPDH
SETP2
FBLN1
ARP2/3
ITGA3B1
GAPDH
Figure 4(c)-2, set1
MYH1
ACTN1
CFL1
GAPDH
Figure 4(c)-2, set2
MYH1
ACTN1
CFL1
GAPDH
Figure 4(c)-2, set3
MYH1
ACTN1
CFL1
GAPDH
Figure 5(a), set1
Ku80
p-p53
p-ATM
Histone H3
p-H2AFX
GAPDH
GAPDH
Figure 5(a), set2
Ku80
p-p53
p-ATM
Histone H3
p-H2AFX
GAPDH
GAPDH
Figure 5(a), set3
Ku80
p-p53
p-ATM
Histone H3
p-H2AFX
GAPDH
GAPDH

Figure S3. All western blot results used for validation of proteomic analysis. The boxed blots (blue) are shown in the main figure.

Table S1. List of cells used in this study.

	Line code	Cell ID	Cell bank	Mutation	Gender	Sample type	Age at sampling	Reprogramming method
**Control**	WT	HPS0076	RIKEN BRC	-	Female	iPSC	36 YR	Episomal vector
**Parkinson's disease**	LK2GS#1	ND38262	Coriell Institute for Medical Research	LRRK2 G2019S	Male	Fibroblast	60 YR	Episomal vector
	LK2GS#2	ND14317	Coriell Institute for Medical Research	LRRK2 G2019S	Male	B-lymphocyte	53 YR	Episomal vector

Table S2. Lists of primer sequences used in qRT-PCR analysis.

Target	Forward	Reverse
GAPDH	TGC ACC ACC AAC TGC TTA GC	GGC ATG GAC TGT GGT CAT GAG
OCT4	GAG GAG TCC CAG GAC ATC AA	AAT AGA ACC CCC AGG GTG AG
NESTIN	CAG GAG AAA CAG GGC CTA CA	TGG GAG CAA AGA TCC AAG AC
PAX6	GTC CAT CTT TGC TTG GGA AA	TAG CCA GGT TGC GAA GAA CT

Table S3. List of antibodies used in western blotting and immunocytochemistry.

Antibodies.	Catalog #	Company	Titer
Established iPSC markers (ICC)			
Anti-OCT4	sc-9081	Santa Cruz Biotechnology, Inc.	1:500
Anti-NANOG	AF1997	R&D Systems	1:40
Differentiated NSC markers (ICC)			
Anti-PAX6	PRB-278P	Covance	1:500
Anti-SOX2	MAB4343	EMD Millipore	1:200
Anti-NESTIN	839801	Biolegend	1:200
Differentiated neuron markers (ICC)			
Anti-TH	T-1299	Sigma-Aldrich	1:500
Anti-MAP2	Ab5392	Abcam	1:5000
Proteomics validation (WB)			
Anti-FBLN1	sc-25281	Santa Cruz Biotechnology, Inc.	1:1000
Anti-ITGA3B1	Ab217145	Abcam	1:500
Anti-ITGB1	Ab52971	Abcam	1:1000
Anti-TLN1/2	sc-365875	Santa Cruz Biotechnology, Inc.	1:1000
Anti-ARP2/3	MABT95	Sigma-Aldrich	1:1000
Anti-CFL1	sc-53935	Santa Cruz Biotechnology, Inc.	1:1000
Anti-PRDX2	sc-515428	Santa Cruz Biotechnology, Inc.	1:1000
Anti-PRDX5	sc-133072	Santa Cruz Biotechnology, Inc.	1:1000
Anti-PRDX6	Ab5790	Abcam	1:1000
Anti-HSP27	sc-13132	Santa Cruz Biotechnology, Inc.	1:1000
Anti-HSP105	sc-74550	Santa Cruz Biotechnology, Inc.	1:1000
Anti-HSPA4	sc-66048	Santa Cruz Biotechnology, Inc.	1:1000
Anti-MYH11	sc-6956	Santa Cruz Biotechnology, Inc.	1:1000
Anti-SEPT2	sc-514206	Santa Cruz Biotechnology, Inc.	1:1000
Anti-ACTN1	sc-17829	Santa Cruz Biotechnology, Inc.	1:2000
Anti-XRCC5	sc-5280	Santa Cruz Biotechnology, Inc.	1:1000
Anti-p-ATM (S1981)	sc-47739	Santa Cruz Biotechnology, Inc.	1:1000
Anti-p-H2AFX (S139)	80312	Cell Signaling Technology (CST)	1:1000
Anti-p-p53 (S15)	82530	Cell Signaling Technology (CST)	1:1000
Anti-Histone H3	14269	Cell Signaling Technology (CST)	1:1000
Anti-GAPDH	sc-47724	Santa Cruz Biotechnology, Inc.	1:5000
Western blot			
Anti-p-LRRK2 (S935)	Ab133450	Abcam	1:1000
Anti-LRRK2	Ab133474	Abcam	1:2000

Anti-Actin	A5441	Sigma-Aldrich	1:2000

Table S4. List of the top10 proteins with the most changes between WT-pNSC and LK2GS-pNSC.

Accession No.	Protein Name	MW (Da)	P*i* Value	Coverage (%)	Matched Peptides	Score		Fold Change (LK2GS/WT)
						WT	LK2GS	
Q9Y2R4	Probable ATP-dependent RNA helicase DDX52 (DDX52)	67.5	9.67	2.5	1	0.00	18.78	LK2GS unique
Q9NXR1	Nuclear distribution protein nudE homolog 1 (NDE1)	38.8	5.27	8.96	2	17.94	0.00	WT unique
Q9Y224	UPF0568 protein C14orf166 (CN166)	28.1	6.65	28.69	5	17.55	0.00	WT unique
P54727	UV excision repair protein RAD23 homolog B (RD23B)	43.1	4.84	4.65	1	14.69	0.00	WT unique
P08133	Annexin A6 (ANXA6)	75.8	5.6	9.96	4	14.63	0.00	WT unique
Q04760	Lactoylglutathione lyase (LGUL)	20.8	5.31	10.87	1	14.52	0.00	WT unique
O43491	Band 4.1-like protein 2 (E41L2)	112.5	5.44	1.09	1	14.49	0.00	WT unique
Q9BQ52	Zinc phosphodiesterase ELAC protein 2 (RNZ2)	92.2	7.9	2.3	1	14.33	0.00	WT unique
P36776	Lon protease homolog, mitochondrial (LONM)	106.4	6.39	6.15	3	13.98	0.00	WT unique
O14980	Exportin-1 (XPO1)	123.3	6.06	3.92	3	13.08	0.00	WT unique
